# Sex-specific digestive performance of mussels exposed to warming and starvation

**DOI:** 10.3389/fphys.2022.991098

**Published:** 2022-09-06

**Authors:** Yueyong Shang, Shuaishuai Wei, Xueqing Chang, Yiran Mao, Sam Dupont, James Kar-Hei Fang, Menghong Hu, Youji Wang

**Affiliations:** ^1^ International Research Center for Marine Biosciences, Ministry of Science and Technology, Shanghai Ocean University, Shanghai, China; ^2^ Key Laboratory of Exploration and Utilization of Aquatic Genetic Resources, Ministry of Education, Shanghai Ocean University, Shanghai, China; ^3^ Department of Biological and Environmental Sciences, The Sven Lovén Centre for Marine Infrastructure, University of Gothenburg, Gothenburg, Sweden; ^4^ Department of Applied Biology and Chemical Technology, The Hong Kong Polytechnic University, Kowloon, Hong Kong SAR, China

**Keywords:** sex difference, mussel, warming, starvation, digestive enzyme

## Abstract

As global climate change has dramatically impacted the ocean, severe temperature elevation and a decline in primary productivity has frequently occurred, which has affected the structure of coastal biomes. In this study, the sex-specific responses to temperature change and food availability in mussels were determined in terms of digestive performance. The thick-shelled mussels *Mytilus coruscus* (male and female) were exposed to different temperature and nutritional conditions for 30 days. The results showed that the digestive enzymes of mussels were significantly affected by temperature, food, sex, and their interactions. High temperature (30°C) and starvation significantly decreased amylase, lysozyme, and pepsase activities of female mussels, while trypsin and trehalase did not change significantly at the experimental end. The activity of amylase, trypsin, and trehalase was significantly reduced in males at high temperature (30°C) under starvation treatment, but high temperature (30°C) elevated pepsase. Unsurprisingly, starvation caused the reduction of lysozyme and pepsase under 25°C in males. Amylase, lipase, and trehalase were higher in female mussels compared with males, while the enzymatic activities of lysozyme, pepsase, and trypsin were higher in male mussels than females. Principal component analysis showed that different enzyme activity indexes were separated in male and female mussels, indicating that male and female mussels exhibited significantly different digestive abilities under temperature and food condition change. The study clarified sex-specific response difference in mussel digestive enzymes under warming and starvation and provided guidance for the development of mussel aquaculture (high temperature management and feeding strategy) under changing marine environments.

## 1 Introduction

The continued emission of greenhouse gasses by human activities from the industrial revolution has subsequently resulted in the increase of atmospheric CO_2_ and global warming. More than 90% of the excess heat stored in the atmosphere is absorbed by oceans, which is mainly responsible for the warming of the ocean (Bindoff et al., 2019; Cheng et al., 2021). A set of measurements of ocean heat content (OHC) in 2020 indicated that global upper 2000 m OHC from 1958 to 2020 has increased with a mean rate of 5.7 ± 1.0 ZJ yr^−1^ (Cheng et al., 2021). Since 1980s, OHC has rapidly increased, with each decade being warmer than the previous decade (Cheng et al., 2021). The conjunction of atmospheric and oceanographic processes caused marine heatwaves, which defined as long-term high surface temperature of ocean (Hobday et al., 2016; He et al., 2021). Ongoing ocean climate warming imposed more challenges of shallow-water ectotherms, such as shellfish, being exposed to beyond-optimal growth temperatures (Vajedsamiei et al., 2021). Lassoued et al. (2021) pointed out that the impact of temperature on the metabolism and growth of marine organisms is an important driving stressor for their adaptation and warming of ocean would affect all their physiological activities to a large extent (Lassoued et al., 2021).

Marine heat wave not only affects the antioxidative responses (He et al., 2021), thermo tolerance (Xu et al., 2022), metabolic performance, and neural function of bivalves (Morosetti et al., 2020; Matoo et al., 2021) but also is an important factor affecting the activity of digestive enzymes. A distinct seasonal pattern of variation has been observed for amylase, protease, and cellulase activities in the digestive gland of the cockle (*Cerastoderma edule*) with maximum digestive enzyme activities in spring and summer (Ibarrola et al., 1998). Khan et al. (2020) showed that warming reduced the activities of amylase, lipase, trypsin, and trehalase in mussels and impaired their digestive functions. The activities of lipase, trypsin, and amylase of the whelk (*Rapana venosa*) also were influenced by water temperature, and lipase activity at 16°C was significantly higher than at 28°C in the digestive gland (Yang et al., 2019). In the context of aquaculture, high temperature can reduce net food intake and body weight of mussels and increase metabolic demands and mortality, resulting in a decrease in fishery productivity (Melzner et al., 2020; Clements et al., 2021). Marine species may show different reproductive cycles due to different water temperature and nutrient conditions (Adzigbli et al., 2019). However, the specific responses of male or female mussels to temperature have received limited attention and sex difference of mussels is always ignored.

Environmental nutrient availability is a key factor in determining physiological traits and the growth rate (Prieto et al., 2018). There are two possible causes of starvation in mussels: insufficient food supply caused by lack of nutrients and suppressed diet due to abnormal temperature and toxins in habitat. For example, generally fewer phytoplankton biomass and less nutrients combined with increased water viscosity could limit filter feeding performance of mussels (Riisgård and Larsen, 2007). Previous studies demonstrated that starvation can affect animal digestive enzymes, energy metabolism, immune responses, antioxidant stress, and reproduction (Ibarrola et al., 1999; Albentosa and Moyano, 2008; Zhang et al., 2010). During starvation, mussels would utilize energy reserves (carbohydrates, lipids, and proteins) from digestive glands and mantle tissues and reduce their metabolic rate *via* increased anaerobic metabolism and decreased oxygen consumption (Bayne, 1973). Starvation can also lead to protein metabolism disorder in mussels (Bayne, 1973). Elevated temperatures often coincide with food shortage due to limiting the vertical exchange of nutrients (Des et al., 2020) and feeding patterns of mussels.

When environmental stressors exist together, adverse impacts will be enhanced (Xie et al., 2021). Khan et al. (2020) found that ocean warming and other factors (e.g., ocean acidification and hypoxia) can lead to compound stress, causing a greater impact on marine organisms than a single factor alone. As for the influence of food supply on mussels under temperature changes, both the mussels’ metabolic demand and food consumption increased strongly at higher temperatures. Under warming conditions, mussels require more energy intake; indeed, a recent study found warmer seawater increased the blue mussels feeding rate during the winter (Melzner et al., 2020). However, the increase of surface temperature may affect the stratification of seawater and limit the vertical exchange of nutrients, thus suppressing the growth of mussels (Des et al., 2020). In order to cope with the high critical temperature, ectotherms in shallow sea may temporarily stop energy-demanding activities such as feeding and growth and then reduce aerobic respiration, so as to avoid high demand for energy and metabolic substrates through metabolic inhibition (Vajedsamiei et al., 2021). For mussels, how their digestive physiology responds to high temperature and food shortage still needs to be studied in depth.

The thick-shelled mussel *M. coruscus* is widely distributed in the southeast coast of China and cultured as typical economic species. Although mussels have been used effectively as indicators of different environmental changes, little information is available on whether physiological responses differ between sexes in thick-shelled mussels. In recent years, aquatic physiology has begun to pay attention to sex differences in the effects of endocrine disrupting pollutants, and studies have shown that different sexes of the same marine shellfish (*Crassostrea angulata*) have different responses to the toxicity of pollutants (Luo et al., 2017a; Luo et al., 2017b). For example, the male snails spent much more time growing under medium food conditions than females (Merot and Collin, 2012), indicating that sex differences existed in the physiological activity of mollusks. In addition, Ji et al., 2014 used a metabolomic analysis and found that female mussel *Mytilus galloprovincialis* was more sensitive to environmental stress than male. However, most experiments in shellfish ecology ignore the sex difference of female and male individual effect. The importance and necessity of food and optimum temperature for organisms is self-evident. Though the impact of warming on bivalves has been studied a lot, the impact on digestive function of different sex mussels under combined impact of thermal stress and starvation has not been clarified. How male and female mussels respond to the change of food conditions and temperatures in the breeding period under the premise of ensuring the development of gonads is a question to be revealed. Therefore, in this study, the digestive performance in terms of digestive enzymes of mussels with different sexes under the combined stress of warming and food shortage was discussed. This study will provide basic information for different strategies of mussels in response to environmental changes during breeding period and assess the health condition of mussel with different sexes under multiple environmental stressors.

## 2 Materials and methods

### 2.1 Experimental animals

Mature mussels *M. coruscus* (shell length 8.8 ± 0.6 cm and wet weight 51.5 ± 4.3 g) were collected from Gouqi Island, Zhejiang Province, China (30^◦^ 43′ 14.268″ N, 122^◦^ 47′ 0.3696″ E) in December 2021, and this sampling time was close to the breeding stage. In total, 500 mussels were handled according to the criterion on the care and use of experimental animals for scientific objective, which was formulated by the Institutional Animal Care and Use Committee (IACUC) of Shanghai Ocean University. The mussels without shell damage were selected and attachments (barnacle and biofouling) on the shells were removed softly. Then those mussels were cultured in laboratory conditions for 14 days to acclimate to the lab environment. Throughout the acclimation phase, the living conditions of mussels were similar to the sampling place: 12-h light:12-h darkness, temperature 20 ± 0.5°C, salinity 25 ± 0.7, and pH 8.1 ± 0.1. The experiment was equipped with the 240 L/h recirculating aquarium system and a gas pump to avoid the influence of waste from mussel metabolism. Throughout the acclimation period, mussels were fed with the microalgae *Chlorella vulgaris* (concentration: 2.5 × 10^4^ cells/mL) twice a day.

### 2.2 Experimental design

To study the effects of temperature change and food conditions as well as their interactions on mussels *M. coruscus* with different sexes, we chose 20, 25, and 30°C combined with two feeding conditions (no feeding and feeding) as experimental conditions for 30 days. Among them, 20°C was set for normal temperature (average sea surface temperature) and 25 and 30°C were set as high temperatures, which are within the range of current summer season variability in the Gouqi Island region (20–30°C) (Shen et al., 2006). In addition, two feeding conditions were set: starvation (no feeding) and feeding groups (fed twice a day with 2.5 × 10^4^ cells/mL *C. vulgaris*), and feeding thick-shelled mussels satiates without producing pseudofaeces at microalgae. After diluting with sterilized seawater and counting by a Coulter counter, 2.5 × 10^4^ cells/mL concentrated algae solution was added twice (daily 6:00 and 18:00) to the feeding group tanks. For each experimental treatment, 20 mussels were cultured in a recirculating tank (30 L), three tanks were used as three replicates (totally 60 mussels), and each individual mussel was considered as a biological replicate. The feces and food residues in each aquarium were cleaned, and the artificial seawater was 100% exchanged weekly. Throughout the experiment process, the number of dead mussels was calculated. On the last day of experiment, the enzymes [including amylase (AMS), lipase (LPS), pepsase (PEP), lysozyme (LZM), trypsin (PS), and trehalase (THL)] related to the digestive performance of digestive glands were tested.

### 2.3 Seawater detection and experimental systems

During the whole experiment, temperature, salinity, and dissolved oxygen were measured by a multifunctional instrument (5200A, YSI Inc., Ohio, United States), and pH was measured by a pH meter (pH 3,310, Germany). The water parameter results are shown in Table 1. The target water temperature of 25 and 30°C was maintained by using constant electronic thermostats, and every tank was wrapped with acrylic plates in order to prevent heat loss and external disturbance.

**TABLE 1:** Chemistry parameters (mean ± SD, *n* = 30) of seawater during the experiment. Temperature (T, ◦C) was measured continuously during the whole experiment. Dissolved oxygen (mg/L), salinity, and pH were measured at every day. Mortality was counted throughout the experiment, and the survival rate (%) was calculated in the end. The sex ratio was determined at the end of experiment (totally 60 mussels in each treatment).

Treatments T (°C)	Salinity	Dissolved oxygen (mg/L)	Temperature (°C)	pH	Survival rate (%)	Sex ratio (Male:Female)
20 × Food	25.0 ± 0.1	6.1 ± 0.3	20.0 ± 0.1	8.10 ± 0.02	100	48: 12
20 × Starvation	25.0 ± 0.3	6.0 ± 0.5	20.0 ± 0.4	8.10 ± 0.03	100	50: 10
25 × Food	24.9 ± 0.2	6.1 ± 0.3	24.9 ± 0.1	8.10 ± 0.01	100	49: 11
25 × Starvation	25.2 ± 0.1	6.2 ± 0.3	24.9 ± 0.3	8.10 ± 0.03	100	53: 7
30 × Food	24.9 ± 0.2	6.0 ± 0.3	30.1 ± 0.2	8.10 ± 0.02	98.3	49: 10
30 × Starvation	24.8 ± 0.4	6.2 ± 0.5	30.1 ± 0.2	8.10 ± 0.02	96.7	50: 8

### 2.4 Sex identification

The sex of mussels was determined by visual inspection of the gonads contained within the mantle tissue (Hines et al., 2007). Color-based identification of the sex is a common method among field studying marine bivalves (Williams & Babcock, 2005). Since the gonads sexually mature male individuals commonly appear cream or milky white color, and female individuals are orange-red color in mussels (Dardignac-Corbeil, 1990). After dissecting, we distinguished the male and female mussel according to the color of gonadal tissue (Dardignac-Corbeil, 1990; Puljas and Brkic, 2020). In the present study, the sex ratios of different treatment groups are shown in Table 1.

### 2.5 Tissue sampling and bioassays

On the last day of exposure, after dissecting the individuals in all treatment groups and identifying the sexes, we randomly selected 10 mussels (five males and five females in each treatment group) from three tanks (in total, 60 mussels was picked out from six treatments) and used sterilized scalpels, scissors, and tweezers to dissect mussels. We used saline (50 mmol/L, pH 7.4, 4°C) to wash digestive glands three times for the purpose of removing attachments and body fluids. Digestive gland samples were dried with a filter paper and then stored in a centrifuge tube and preserved at −80°C for further analysis. Before detection, samples were unfrozen on ice and the activities of different digestive enzymes were determined by using a specific homogenizing method, such as saline solution (AMS, LZM, and LPS) and specific homogenization medium (PEP, PS, and THL). A teflon potter-Elvehjem homogenizer was used to homogenize at 4°C. Homogenate samples were centrifuged (12,000 g, 4°C, 8 min), and then the supernatant liquid was collected for a biochemical analysis. After homogenate, the samples were detected on time to prevent the decrease of enzyme activities.

Commercial assay kits purchased from Nanjing Jian Cheng Bioengineering Research Institute (Nanjing, China) were used for performing assays by following relevant instructions. An automatic microplate reader (Flexstation^®^ 3, Moleclar Devices, California, United States) was used to detect optical density values. The total protein was detected using the Coomassie Brilliant Blue (G-250) method by Bradford (1976).

#### 2.5.1 Amylase

The iodine–starch colorimetry (Valsanescu et al., 1985) was used to measure amylase activity at 660 nm by using an amylase determination kit (product code: C016-1-1). Tissue protein reacts with substrates at 37°C per milligram for 30 min; the hydrolysis of 100 mg starch was defined as a unit of amylase activity (U/mg prot).

#### 2.5.2 Lipase

The methyl halogen substrate method was used to detect lipase activity by using a lipase determination kit (product code: A054-2-1). At 580 nm, the activity of lipase activity was determined according to the formation rate of red methyl trihalide. The lipase activity (U/mg prot) was determined by the production rate of red methyl resorufin with the method of spectrophotometry at a wavelength of 580 nm in 10 min at 37°C.

#### 2.5.3 Lysozyme

Turbidimetry was used to detect lysozyme activity by using a lysozyme determination kit (product code: A050-1-1). Before experimental determination, all samples needed to be incubated in a water bath (37°C) for 15 min. Then the reaction was terminated immediately by an ice bath. The content of lysozyme was detected according to the change of transmittance. At 530 nm wavelength and 37°C, lysozyme is defined as a change in transmittance of 0.001/min.

#### 2.5.4 Pepsin

Pepsin activity can be determined with colorimetry by using a pepsin determination kit (product code: A080-1-1). Breaking down protein at 37°C produces 1 μg of tyrosine per minute equivalent to one unit of enzymatic activity at 660 nm.

#### 2.5.5 Trypsin

Trypsin activity was calculated according to the change in absorbance at 253 nm by using a trypsin determination kit (product code: A080-2-2). At 37°C and pH 8.1, 0.003 value change in absorbance in per milligram of protein was used as the unit of trypsin.

#### 2.5.6 Trehalase

The spectrophotometric method was used to detect trehalase activity by using a trehalase determination kit at 505 nm (product code: A150-1-1). The catalytic production of 1 mmole of glucose per milligram of protein per minute is defined as one unit of trehalase enzymatic activity.

### 2.6 Statistical analyses

The data were tested for normality and homogeneity of variance by Shapiro–Wilk’s test and Levene’s test in Statistical Product and Service Solutions 22.0 (SPSS Inc., Chicago, IL, United States), respectively. Then temperature, food, and sex were used as independent variables by SPSS for three-way ANOVA. For digestive parameters, if an interaction was observed, one-way ANOVA and Student’s *t*-test were used to analyze whether temperature, food, or sex had a significant effect, with *p* < 0.05 as a significant difference. The statistical data was denoted by mean ± standard deviation (Mean ± SD). In addition, GraphPad Prism 6 and Origin 2018 were used for drawing histograms and principal component analysis plot, respectively.

## 3 Results

### 3.1 Survival and sex ratio

Elevated temperature (25°C) did not affect the survival of mussel, but high temperature (30°C) caused individual death (Table 1). Under high temperature treatment treatments, one mussel died in the food treatment group and two died in the starvation group. In addition, starvation had no significant impact on survival. The sex ratio of different treatment groups is shown in Table 1. Both survival and sex ratio among different treatments were not significantly different.

### 3.2 Digestive enzyme

**FIGURE 1:**
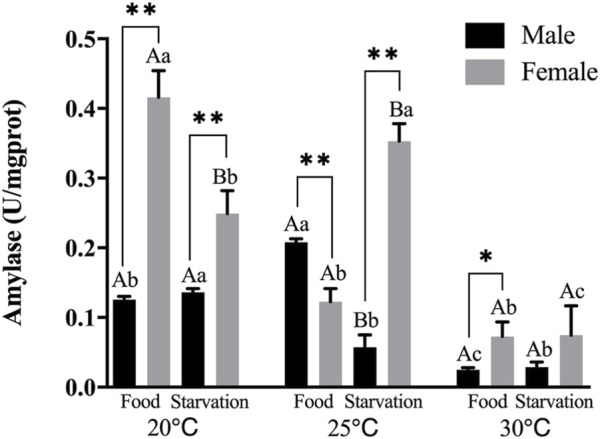
Amylase (AMS) of different sex *M. coruscus* exposed to six combinations of temperature (20, 25, and 30°C) and feeding condition (food and starvation) for 30 days during the exposure period. Different capital letters indicate significant differences between the feeding condition and each temperature levels (20, 25, and 30°C) (*p* < 0.05). Different small letters indicate significant differences between temperature (20, 25, and 30°C) and the same feeding condition (*p* < 0.05). Asterisk indicates significant differences between sex and each feeding condition, in which * represents significant difference (*p* < 0.05) and ** represents highly significant difference (*p* < 0.01).

Feeding condition, sex, and temperature had significantly affected amylase (AMS) activity throughout the experiment, and there were significant interactions among temperature, food, and sex (*p* < 0.01, Table 2). For the effect of temperature, significant high AMS was observed at high temperature (25°C) in Male-Food treatment and significant low AMS activity was observed at high temperature (30°C) in Male-Starvation treatment. AMS was significantly decreased due to high temperature in Female-Food treatment and also significantly decreased at 30°C under starvation condition (Figure 1). For the effect of food condition, starvation significantly reduced AMS of male at 25°C, but starvation did not affect AMS in male treatment at high temperature (30°C). Similarly, AMS of female was not significant affected by starvation at 30°C (Figure 1). In addition, sex differences were significantly noted by feeding condition, except 25°C-Food treatment, AMS activity of female was significantly higher than male (Figure 1).

**TABLE 2:** Summary of three-way ANOVA results on effects of temperature (T), food (F) and sex (G) on amylase (AMS), lipase (LPS), lysozyme (LZM), pepsase (PEP), trypsin (PS), and trehalase (THL).

	Df	AMS			LPS			LZM			PEP			PS			THL		
		MS	F	P	MS	F	P	MS	F	P	MS	F	P	MS	F	P	MS	F	P
T	2	0.107	207.810	<0.01	84.392	175.261	<0.01	15.625	87.690	<0.01	56.693	39.879	<0.01	6155457.575	35.695	<0.01	1,9782.663	79.916	<0.01
F	1	0.001	2.450	0.131	0.623	1.293	0.267	0.016	0.090	0.766	11.072	7.789	0.010	3070815.863	17.807	<0.01	562.006	2.270	0.145
G	1	0.125	244.217	<0.01	1,240.551	2,576.326	<0.01	4.343	24.371	<0.01	219.755	154.581	<0.01	61576077.305	357.076	<0.01	0.550	0.002	0.963
T*F	2	0.011	21.359	<0.01	83.004	172.379	<0.01	0.884	4.960	0.016	4.113	2.893	0.075	4454761.884	25.833	<0.01	1,0774.776	43.527	<0.01
T*G	2	0.018	35.853	<0.01	115.935	240.769	<0.01	3.557	19.964	<0.01	29.313	20.620	<0.01	3544379.564	20.554	<0.01	3,0300.088	122.403	<0.01
F*G	1	0.010	19.909	<0.01	16.766	34.819	<0.01	3.723	20.893	<0.01	41.897	29.471	<0.01	38,8576.131	2.253	0.146	716.098	2.893	0.102
T*F*G	2	0.061	119.233	<0.01	52.855	109.767	<0.01	5.162	28.968	<0.01	62.830	44.196	<0.01	5627791.846	32.635	<0.01	1,111.068	4.488	0.022

**FIGURE 2:**
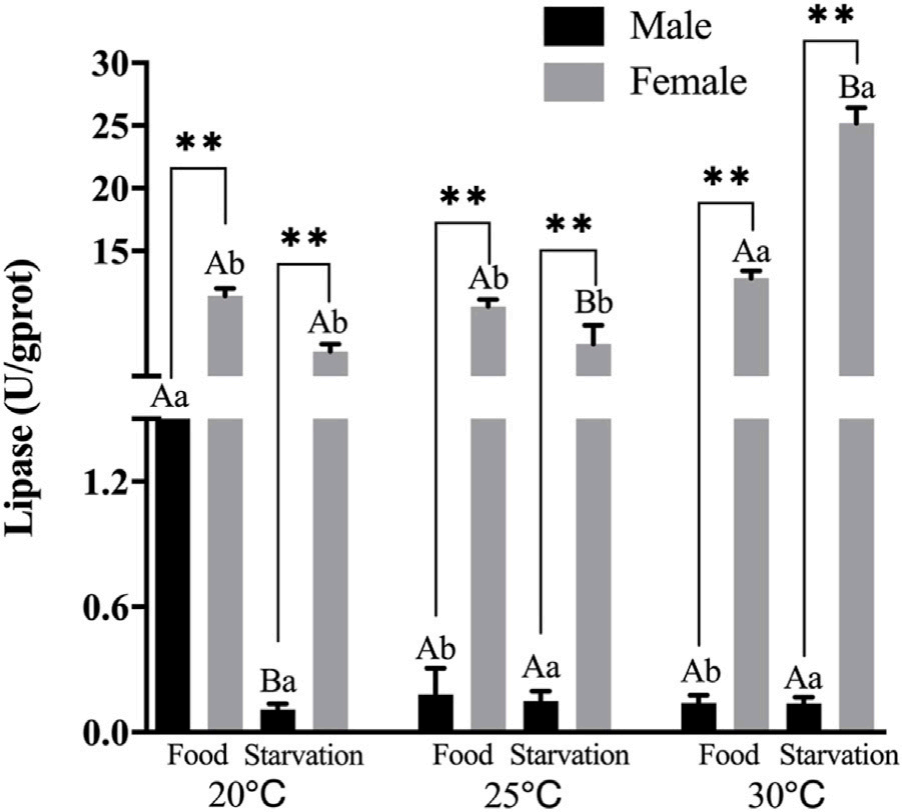
Lipase (LPS) of different sex *M. coruscus* exposed to six combinations of temperature (20, 25, and 30°C) and the feeding condition (food and starvation) at 30 days during the exposure period. Different capital letters indicate significant differences between the feeding condition and each temperature levels (20, 25, and 30°C) (*p* < 0.05). Different small letters indicate significant differences between temperature (20, 25, and 30°C) and the same feeding condition (*p* < 0.05). Asterisk indicates significant differences between sex and each feeding condition, in which * represents significant difference (*p* < 0.05) and ** represents highly significant difference (*p* < 0.01).

Significant effects of feeding condition, sex, and temperature on lipase (LPS) activity during the experiment were noted, and there were significant interactions among temperature, food, and sex (*p* < 0.01, Table 2). For the effect of temperature, LPS activity of high temperature (25 and 30°C) was significantly lower than normal temperature in Male-Food condition, but high temperature had no significant impact on LZM in the starvation condition. Compared to normal temperature, 25°C did not have significant impact on LPS, but high temperature (30°C) significantly increased LPS in female (Figure 2). For the effect of food condition, starvation significantly reduced LPS at normal temperature, but starvation did not affect LPS under high temperature (25 and 30°C) in male. At normal temperature, feeding condition did not have significant effect on LPS; but at high temperature (30°C), starvation significantly increased LPS in female (Figure 2). In addition, sex differences were significantly noted by feeding condition, and LPS of female was significantly higher than male (Figure 2).

**FIGURE 3:**
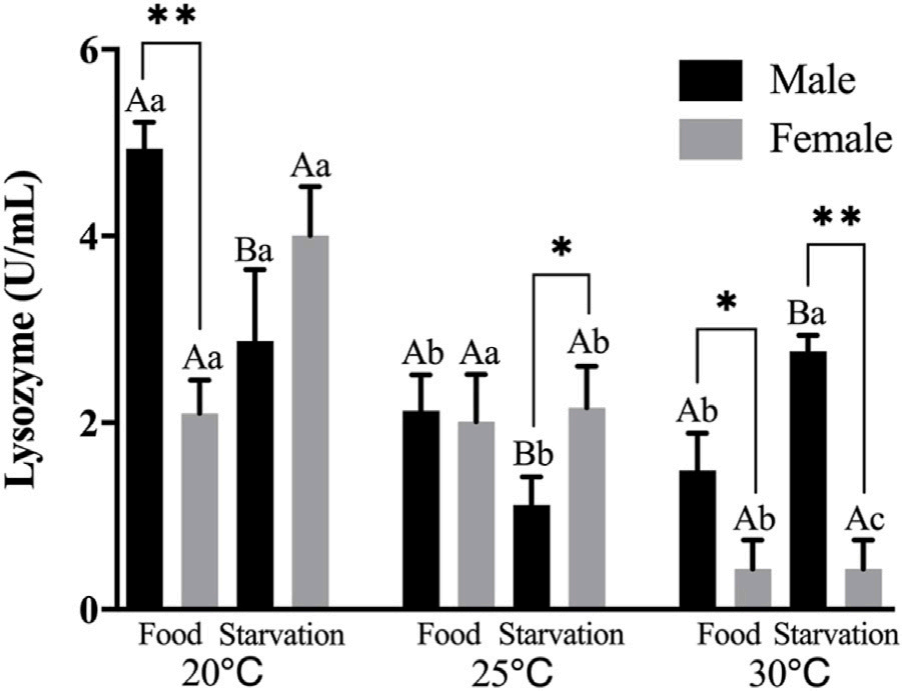
Lysozyme (LZM) of different sex *M. coruscus* exposed to six combinations of temperature (20, 25, and 30°C) and the feeding condition (food and starvation) at 30 days during the exposure period. Different capital letters indicate significant differences between the feeding condition and each temperature levels (20, 25, and 30°C) (*p* < 0.05). Different small letters indicate significant differences between temperature (20, 25, and 30°C) and the same feeding condition (*p* < 0.05). Asterisk indicates significant differences between sex and each feeding condition, in which * represents significant difference (*p* < 0.05) and ** represents highly significant difference (*p* < 0.01).

Lysozyme (LZM) activity was significantly affected by feeding condition, sex, and temperature during the experiment, and there were significant interactions among temperature, food, and sex (*p* < 0.01, Table 2). For the effect of temperature, increased temperature (25 and 30°C) significantly reduced LZM in Male-Food treatment and compared to normal temperature, high temperature (25°C) significantly decreased LZM in Male-Starvation treatment, but there was no significant impact on LZM at 30°C. In addition, increased temperature significantly reduced LZM in female treatments (Figure 3). For the effect of food condition, starvation significantly reduced LZM at normal and high temperature (25°C) in male, but the results of 30°C were opposite. Also, starvation had no significant impact on LZM at high temperature in female treatment (Figure 3). Sex differences were significantly noted by feeding condition; in 20°C-Food treatment and high temperature (30°C) treatment, LZM of male was significantly higher than female (Figure 3).

**FIGURE 4:**
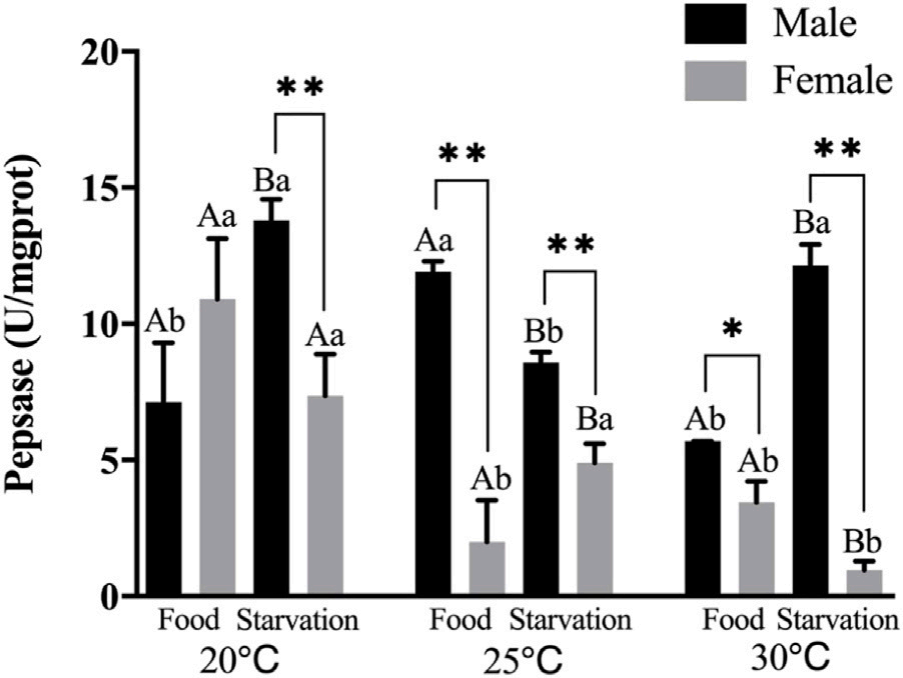
Pepsin (PEP) of different sex *M. coruscus* exposed to six combinations of temperature (20, 25, and 30°C) and the feeding condition (food and starvation) at 30 days during the exposure period. Different capital letters indicate significant differences between the feeding condition and each temperature levels (20, 25, and 30°C) (*p* < 0.05). Different small letters indicate significant differences between temperature (20, 25, and 30°C) and the same feeding condition (*p* < 0.05). Asterisk indicates significant differences between sex and each feeding condition, in which * represents significant difference (*p* < 0.05) and ** represents highly significant difference (*p* < 0.01).

Significant effects of feeding condition, sex, and temperature were noted for pepsase (PEP) activity during the experiment and was affected by a combined effect of them apart from the interaction of temperature and food (*p* < 0.01, Table 2). For the effect of temperature, high temperature (25°C) significantly increased PEP in Male-Food treatment. Increased temperature (25°C) significantly reduced PEP, but high temperature (30°C) did not significantly affect PEP in Male-Starvation treatment. Also, high temperature (25 and 30°C) significantly reduced PEP in female treatments (Figure 4). For the effect of food condition, starvation significantly reduced PEP at 25°C, but it increased at 30°C in male treatment. Also, starvation significantly increased PEP at 25°C, but it decreased at 30°C in female treatment (Figure 4). Sex differences were significantly noted by feeding condition; in 20°C-Starvation treatment and high temperature treatment (25 and 30°C), pepsase activity of male was significantly higher than female (Figure 4).

**FIGURE 5:**
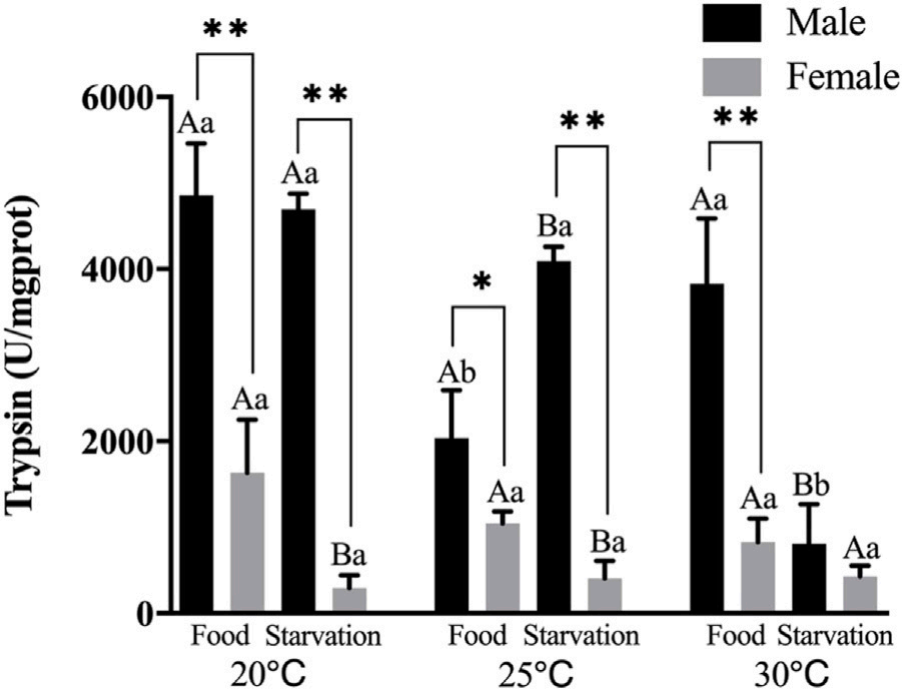
Trypsin (PS) of different sex *M. coruscus* exposed to six combinations of temperature (20, 25, and 30°C) and the feeding condition (food and starvation) at 30 days during the exposure period. Different capital letters indicate significant differences between the feeding condition and each temperature levels (20, 25, and 30°C) (*p* < 0.05). Different small letters indicate significant differences between temperature (20, 25, and 30°C) and the same feeding condition (*p* < 0.05). Asterisk indicates significant differences between sex and each feeding condition, in which * represents significant difference (*p* < 0.05) and ** represents highly significant difference (*p* < 0.01).

Trypsin (PS) activity was significantly affected by feeding condition, sex, and temperature during the experiment and was affected by combined effect of them apart from the interaction of food and sex (*p* < 0.01, Table 2). For the effect of temperature, 25°C-Food treatment significantly decreased PS in male, but high temperature (30°C) had no significant impact on PS. Also, high temperature (30°C) significantly decreased PS in Male-Starvation treatment. In addition, high temperature did not affect PS in female (Figure 5). For the effect of food condition, starvation significantly increased PS at 25°C, but PS was decreased at 30°C in male. Starvation significantly reduced PS at 25°C, and there was no significant effect on PS at 30°C in female (Figure 5). Sex differences were significantly noted by feeding condition, PS of male was significantly higher than female (Figure 5).

**FIGURE 6:**
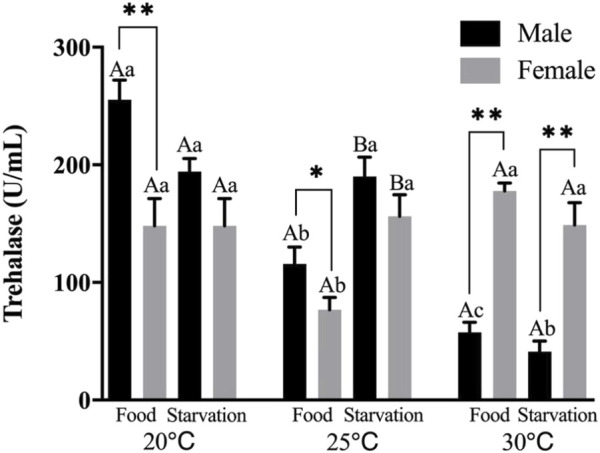
Trehalase (THL) of different sex *M. coruscus* exposed to six combinations of temperature (20, 25, and 30°C) and the feeding condition (food and starvation) at 30 days during the exposure period. Different capital letters indicate significant differences between the feeding condition and each temperature levels (20, 25, and 30°C) (*p* < 0.05). Different small letters indicate significant differences between temperature (20, 25, and 30°C) and the same feeding condition (*p* < 0.05). Asterisk indicates significant differences between sex and each feeding condition, in which * represents significant difference (*p* < 0.05) and ** represents highly significant difference (*p* < 0.01).

Significant effects of feeding condition, sex, and temperature were noted for trehalase (THL) activity during the experiment, which was affected by combined effect of temperature and food as well as temperature and sex (*p* < 0.01, Table 2). For the effect of temperature, high temperature (25 and 30°C) significantly decreased THL in male. The temperature of 25°C significantly decreased THL, but 30°C had no significant effect on THL in Female-Food treatment. Also, high temperature (25 and 30°C) had no significant impact on THL in Female-Starvation treatment (Figure 6). For the effect of food condition, starvation significantly increased THL at high temperature (25°C) in male, but there was no significant difference in normal and high temperature (30°C). Starvation significantly increased THL in female at 25°C, but there was no significant difference in normal and high temperature (30°C) (Figure 6). Sex differences were significantly noted by feeding condition. In 20 and 25°C-Food treatments, THL activity of male was significantly higher than female but significantly lower than female at 30°C (Figure 6).

### 3.3 Principal component analysis

**FIGURE 7:**
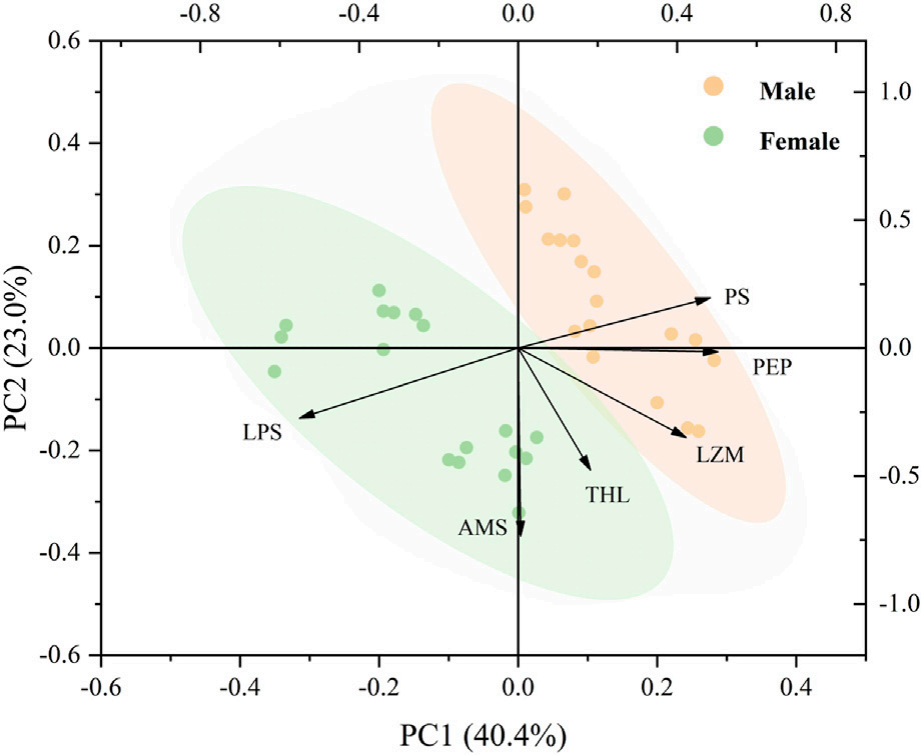
Biplot originating from PCA integrating all measured variables (AMS, LPS, LZM, PEP, PS, and THL) and three temperature levels (20, 25, and 30°C) at six different treatments during the exposure period.

The PCA of the effect of temperature, food, and sex on the digestive enzymes showed that the two main components accounted for 63.4% of the total composition (Figure 7). PC1 accounted for 40.4% of the total variance, and the horizontal axis represents the specific responses of females and males, as well as the individual responses of males and females on digestive enzyme parameters, indicating a significant difference in the digestive ability of male and female mussels. LPS and AMS were negatively correlated with PS, PEP, LZM, and THL (Figure 7).

## 4 Discussion

With the change of season, the activity and composition of digestive enzymes of mollusks change to a certain extent (Gao et al., 2009; Charron et al., 2013). Seasonal change is mainly manifested in ambient temperature (Nie et al., 2016) and composition of different food (Reid and Rauchert, 1976), among which temperature has the most direct and greatest influence on digestive enzymes. Digestive enzymes are secreted by the digestive system, breaking down large food molecules into smaller components that can be absorbed by organisms, to maintain growth, breeding, and other activities that are needed to sustain life energy and nutrition of shellfish. Digestive enzyme activity of shellfish is an important index reflecting the digestive physiology ability (Fu et al., 2005), which is greatly related to environmental parameters. Enzymes are proteins, so temperature is critical to their biochemical activity and stability. Each enzyme has its own specific biochemical structure, and small changes in these factors can adversely affect the catalytic function of the enzyme (Khan et al., 2020). By increasing of organic components in experimental diets, the digestive gland of the common cockle *Cerastoderma edule* would increase its size as well as specific cellulase activity to induce higher enzyme activities (Ibarrola et al., 1998). Adequate or inadequate food conditions would lead to changeable digestive enzyme activities. The relationship between food availability and short-term changes in digestive enzyme activity suggests that the seasonal pattern of enzyme activity reflects the continuous short-term adjustment process of enzyme levels to seasonal changes in food availability. However, the influence of environmental factors on biological responses has been the focus of research, such as temperature (Charron et al., 2013) and salinity (Sornom et al., 2010). Although the influence of external factors has been well studied, sex differences have not been well explored. In general, sex as a biotic parameter (Anantharaman and Craft, 2012) also must be thought to be factors that may influence biomarkers. In the present study, after 30-day exposure period, the digestive enzyme activities of mussels were significantly changed, especially the sex difference under the stress of warming and food change, and the results implied that male and female mussels adopt different food digestion strategies to cope with multifactorial environmental stress.

During the experimental process, the mussels from high temperature treatments (30°C) showed a few individual death, but 25°C and starvation did not affect the survival of mussels during 30 days exposure, indicating the thick-shelled mussels can adapt the change of temperature and food availability to some degree. Albentosa and Moyano, 2008 found that food deprivation results in a sharp reduction in all digestive enzyme activities (Albentosa and Moyano, 2008). In spite of such an obvious reduction, reaching almost 80% in some cases, the basic levels of various activities remained after 15 days of food deprivation. This suggests that mussels would adjust their energy supply and physiological activity in response to the negative effects of environmental changes when food is in short supply. This is consistent with the present experimental results that starvation did not affect survival of mussels.

Amylase is one of the main digestive enzymes in the process of food metabolism. It hydrolyzes amylose and glycogen to provide materials for the growth and development of shellfish, which is an important physiological parameter affecting the digestion ability (Inomata et al., 1995). It was found that temperature and other environmental factors could affect amylase activity of aquatic organisms. Gao et al. (2009) found that low temperature (7°C) did not significantly affect amylase compared to average sea surface temperature (21°C), but amylase was significantly decreased by increased seawater temperature (28°C) in sea cucumber *Apostichopus japonicus* (Gao et al., 2009). Compared to other exposure treatments, high temperature (30°C) significantly reduced amylase in present experiment, which is similar to aforementioned study. Furthermore, two marine clam species *Ruditapes decussatus* and *Venerupis pullastra* showed obvious sensitivity of amylase after 2 weeks starvation; the amylase respectively accounted for 30% and 20% of total carbohydrase activity, which accounted by 50% and 30% before exposure, respectively (Albentosa and Moyano, 2008). Compared to feeding groups, amylase was not significantly affected by food deprivation at high temperatures (30°C) in our study. However, high temperature decreased the activity of amylase in food and starvation treatments compared to normal condition, which could be that high temperature restricted the ability of ingestion in mussel under an abundance of food condition. Therefore, under warming condition, food abundance could not induce the activity of amylase. When exposed to 25°C, starvation significantly reduced amylase in male treatment. The relationship of declined amylase activity and starvation could reflect the energy optimization strategies under reduced dietary intake (Charron et al., 2014). In addition, the effect of temperature and food on amylase of mussel showed obvious sex difference. Under starvation, the amylase activity of male was significantly lower than female. This may be related to female gonadal development, where exogenous yolk was produced in large quantities (Jiang et al., 2012) and therefore required sufficient amylase to absorb exogenous nutrients.

Lipase exists widely in aquatic animals, which is an enzyme with many kinds of catalytic ability, such as catalyzing the decomposition of chylomicrons and triglycerides (Masuno et al., 1991). Khan et al. (2020) indicated that lipase activity significantly reduced when seawater temperature rose from 20 to 30°C, suggesting the digestive performance of *M. coruscus* was impaired at 30°C, which was similar with the present study of male treatments. Gao et al. (2009) also observed that lipase was significantly decreased by high seawater temperature in sea cucumber *A. japonicus*. High lipase activity of *Rapana venosa* was detected at 16°C, which was obviously higher than those at 28°C (Yang et al., 2019). In contrast, there is no significant effect of lipase when mussel exposure to elevated seawater temperature (25°C) in female treatment, but high temperature (30°C) significantly increased lipase activity, showing significant sex difference. Temperature and starvation damaged the activity of lipase, however, there is no significant difference in female at 25°C. Meanwhile, we found significantly increased lipase activity at 30°C in female. This may have something to do with the energy supply of female mussels during breeding. For example, during the crustacean reproductive cycle, many studies demonstrated that breeding female would store lipids (Sutcliffe, 2010). This phenomenon indicated that under extreme conditions (warming or starvation), female mussels with adequate lipids store may be more resistant to stress than male mussels.

Lysozyme is a typical humoral defense component, a large number of experiments have studied its role and mechanism in the immune response of marine bivalves (Muroga and Takahashi, 2007; Hu et al., 2015; Sun et al., 2022). The action mechanism of lysozyme mainly lies in its ability to destroy peptidoglycan components in dissolved bacterial cell wall, thus causing bacterial cell wall damage and disintegration (Liu et al., 2003). Hu et al. (2015) indicated that pH, temperature, dissolved oxygen and exogenous objects can easily change the activity of lysozyme. Speaking of the temperature effect, lysozyme activities at 23°C were obviously higher than high temperature of 33°C (Sun et al., 2022). In this study, with the increase of temperature, lysozyme activity was significantly reduced, which is similar to the above study. In addition, during the 30 days of high temperature treatment, the lysozyme activity of the triangle sail mussel *Hyriopsis cumingii* showed a trend of increase first and then a decrease, indicating if mussels were exposed to such environment for a long term, the immune capacity of digestive gland would be damaged (Sun et al., 2022). Digestive enzyme activities (Albentosa and Moyano, 2008) and immune enzyme activities (Mahapatra et al., 2017) were decreased after exposure to semi-starvation treatment. Sun et al. (2022) reported that compared to excessive dietary and semi-starvation, lysozyme activities were significantly higher in satiation treatment. Similarly, our results demonstrated that starvation reduced the activity of lysozyme under normal and high temperature condition (25°C) in male treatments. In addition, during the high temperature (30°C) exposure, lysozyme showed significant sex difference and lysozyme activity in male was significantly higher than female. As a biomarker of health condition and the vitality of defense performance in bivalves, the changing trend of lysozyme was consistent with other enzymes (eg., trypsin and pepsin) and kept a stable status all the time, suggesting female mussels have a better tolerance to high temperatures.

Pepsin is a digestive protease that breaks down proteins in food into small peptide fragments. Our experimental results demonstrated that compared to normal temperature (20°C), high temperature reduced the activities of pepsin. Starvation also affected pepsin activities. Pepsin activity showed a decreased trend after prolonged starvation in the red swamp crayfish *Procambarus clarkia* (Chen et al., 2018). In the same species, Lu et al. (2012) also found reduced pepsin activity under starvation, which is similar to our results. In addition, the effect of temperature and food on pepsin of mussels showed obvious sex differences. The lower digestive enzyme activity was found in female mussels might imply that female mussels have a lower consumption rate than male mussels when they faced food shortage, since digestive enzyme activity is usually associated with food consumption rates (Areekijseree et al., 2004). According to this study, when food is scarce, female mussels reduced their energy supply to the proteins in the digestive glands and may redirect energy to the gonads during reproduction.

Seasonal changes of seawater temperature influenced the trypsin storage contents in digestive tissues under starvation (Kofuji et al., 2010). Khan et al. (2020) found that trypsin activity was negatively correlated with temperature, and significantly decreased with temperature rising from 20 to 30°C in *M. coruscus*. In sea cucumber, elevated seawater temperature also significantly decreased trypsin activities (Gao et al., 2009). In our experiment, increasing temperature had no significant effects on trypsin, especially in female treatments. There is significant sex difference of trypsin, and the activities of male obviously higher than female during the whole process. Previous study indicated that enzyme activities can reflect a process of continuous modulation of digestive performance to environmental variation (Ibarrola et al., 1998). This indicated that trypsin is not the primary digestive enzyme used to maintain physiological activity when female mussels were exposed to high temperature and food shortage stress.

Trehalase exists widely in animals and can decompose trehalose in the body to generate glucose for energy supply (Shukla et al., 2016). Trehalose acts as a stress protectant, and its accumulation protects body from oxidation, freezing and heat (Bao et al., 2021). In a previous study, high temperature significantly decreased trehalase activities and exposure time could exacerbate this negative effect of mussels (Khan et al., 2020). It is similar to the present study that high temperature reduced the activity of trehalase in male. The reduction of trehalase activities indicated that energy was allocated to growth and recovery from abiotic stress would be inhibited (Shukla et al., 2016). In addition, the effect of temperature and food on trehalase of mussel showed obvious sex difference. Although, the activity of female was lower than male in normal and high temperature (25°C), temperature and food shortage did not have obvious effect on trehalase in female during the whole experimental period, indicating the trehalase activity of female mussels did not fluctuate significantly with the change of environment, but remained at a relatively stable level.

## 5 Conclusion

Ectotherms may respond to high temperatures by temporarily stopping energy-demanding activities such as feeding and growth. According to our results, high temperature (30°C) shaved digestive enzyme activities substantially, which was synergistic with starvation. In addition, the sex difference between male and female mussels produced different physiological responses to environmental changes. Although the activity of some digestive enzymes (e.g., trypsin and pepsin) were more active in male mussel, but fluctuating by temperature and food conditions easily, whereas the activity of these enzymes remained relatively stable in female mussels. Those results indicated that female mussels were more resistant to environmental stress than male mussels. Due to that digestive enzyme activities are usually associated with food consumption rates, the different enzyme activities may indicate that male or female will prioritize different energy sources when they face environmental stress. Our results give vital data for health assessment of mussels in breeding season under warming and starvation and provide new insights to studies on mussel aquaculture and ecology.

## Data Availability

The original contributions presented in the study are included in the article/Supplementary Material; further inquiries can be directed to the corresponding authors.
